# Altered Cerebellar Response to Somatosensory Stimuli in the *Cntnap2* Mouse Model of Autism

**DOI:** 10.1523/ENEURO.0333-21.2021

**Published:** 2021-10-19

**Authors:** Marta Fernández, Carlos A. Sánchez-León, Javier Llorente, Teresa Sierra-Arregui, Shira Knafo, Javier Márquez-Ruiz, Olga Peñagarikano

**Affiliations:** 1 Department of Pharmacology, University of the Basque Country (UPV/EHU), Leioa 48940, Spain; 2 Department of Physiology, Anatomy and Cellular Biology, Pablo de Olavide University, Seville 41013, Spain; 3 Leibniz Research Center for Working Environment and Human Factors, Department of Psychology and Neurosciences, Technical University Dortmund, Dortmund 44227, Germany; 4 Department of Physiology and Cell Biology, The National Institute for Biotechnology in the Negev, and The Zlotowski Center for Neuroscience, Ben-Gurion University of the Negev, Beer-Sheva 8410501, Israel; 5 Centro de Investigación Biomédica en Red de Salud Mental (CIBERSAM), Leioa 48940, Spain; 6 Ikerbasque, Basque Foundation for Science and Instituto Biofisika, Leioa 48940, Spain

**Keywords:** autism, cerebellum, cntnap2, complex spike, Purkinje, sensory stimuli

## Abstract

Atypical sensory processing is currently included within the diagnostic criteria of autism. The cerebellum is known to integrate sensory inputs of different modalities through its connectivity to the cerebral cortex. Interestingly, cerebellar malformations are among the most replicated features found in postmortem brain of individuals with autism. We studied sensory processing in the cerebellum in a mouse model of autism, knock-out (KO) for the *Cntnap2* gene. *Cntnap2* is widely expressed in Purkinje cells (PCs) and has been recently reported to regulate their morphology. Further, individuals with *CNTNAP2* mutations display cerebellar malformations and CNTNAP2 antibodies are associated with a mild form of cerebellar ataxia. Previous studies in the *Cntnap2* mouse model show an altered cerebellar sensory learning. However, a physiological analysis of cerebellar function has not been performed yet. We studied sensory evoked potentials in cerebellar Crus I/II region on electrical stimulation of the whisker pad in alert mice and found striking differences between wild-type and *Cntnap2* KO mice. In addition, single-cell recordings identified alterations in both sensory-evoked and spontaneous firing patterns of PCs. These changes were accompanied by altered intrinsic properties and morphologic features of these neurons. Together, these results indicate that the *Cntnap2* mouse model could provide novel insight into the pathophysiological mechanisms of autism core sensory deficits.

## Significance Statement

Atypical sensory processing is currently included within the diagnostic criteria of autism. The cerebellum is known to integrate sensory inputs of different modalities through its connectivity to the cerebral cortex. In support of this, cerebellar malformations are among the most replicated features found in postmortem brain of individuals with autism. One autism-linked gene associated to cerebellar dysfunction both in humans and animal models is *CNTNAP2*. In this work, we studied cerebellar integration of sensory information in the Cntnap2 mouse model of autism. We found striking differences between wild-type and *Cntnap2* knock-out mice that indicate an altered cerebro-cerebellar connection. In addition, single-cell recordings identified alterations in both sensory-evoked and spontaneous firing patterns of Purkinje cells. These alterations were accompanied by altered intrinsic properties and morphologic features of these neurons. Although the mechanism of such deficits is not revealed, these data indicate that the *Cntnap2* mouse model could be very valuable to identify the pathophysiological mechanisms of autism spectrum disorder core sensory deficits.

## Introduction

Atypical sensory processing is currently included within the diagnostic criteria of autism spectrum disorder (ASD; American Psychiatric Association, 2013). The cerebellum is known to play a role in integration of different sensory modalities (e.g., hearing, sight, touch and smell) through its connectivity to the cerebral cortex (Proville et al., 2014). Interestingly, cerebellar malformations are among the most replicated features found in postmortem brain of individuals with autism (Wang et al., 2014) and alterations in functional connectivity between the cerebellum and cortical sensory areas have been found through fMRI (Lidstone et al., 2021).

Loss of function mutations in the *CNTNAP2* gene are associated with a syndromic form of autism that presents with cerebellar abnormalities, including hypoplasia of the cerebellar vermis and hemispheres (Rodenas-Cuadrado et al., 2016). Further, autism-linked common genetic variation in *CNTNAP2* has been associated with reduction in cerebellar gray matter volume, as determined by MRI (Tan et al., 2010). Incidentally, CNTNAP2 antibodies have been identified in sera from patients with otherwise unexplained progressive cerebellar ataxia with mild to severe cerebellar atrophy (Becker et al., 2012; Melzer et al., 2012), supporting the role of this gene in cerebellar development and function. *CNTNAP2* is widely expressed in the cerebellum (Gordon et al., 2016), and it has been recently reported to regulate Purkinje cell (PC) morphology (Argent et al., 2020). In mice, loss of *Cntnap2* function causes autism-like behaviors (Peñagarikano et al., 2011), as well as several sensory abnormalities such as hypersensitivity to painful stimuli (Dawes et al., 2018) and alterations in auditory (Scott et al., 2018) as well as olfactory behaviors (Gordon et al., 2016). Neuroanatomical analysis of this model shows an alteration in cerebellar volume, as measured by structural MRI (Ellegood and Crawley, 2015). Classical tests measuring cerebellar function report improved performance in the accelerating rotarod (Peñagarikano et al., 2011), unstable gait (Argent et al., 2020), and cerebellar sensory learning defects (Kloth et al., 2015). However, a physiological analysis of cerebellar function has not been performed yet. In this work, we studied sensory processing in the cerebellum in alert *Cntnap2* mice. We discovered alterations in the firing patterns of PC both spontaneous as well as in the evoked response to sensory stimuli. This alteration was accompanied by an increased excitability of PC and reduced dendritic complexity in these neurons. Together, these results provide novel insight into the pathophysiological mechanisms by which *CNTNAP2* mutations cause impairments in cerebellar function that may contribute to ASD core deficits.

## Materials and Methods

### Animals

Adult (8–10 weeks) male mutant mice lacking the *Cntnap2* gene [Cntnap2-knock-out (KO)] and age-matched wild-type (WT) controls (C57BL/6 background) were purchased from The Jackson Laboratory. Animals were housed four to five per cage on a 12–12 h light/dark cycle, at 21–23°C and 65–70% humidity. Food and water were provided *ad libitum*. Animal maintenance and experimental procedures were executed following the guidelines of animal care established by the European Communities Council Directive 2010/63/EU, as well as in agreement with the Spanish Legislation (Royal Decree 53/2013). Procedures were also approved by the Ethics Committee for Animal Welfare (CEBA) of the University of the Basque Country (UPV/EHU) and the Pablo de Olavide University (UPO).

### *In vivo* electrophysiology

We followed the procedures described in Sánchez-León et al. (2021). Briefly, stereotaxic surgery was performed to open a craniotomy (2 mm ø) following the Allen Brain Atlas coordinates for the right Crus I/II area (AP: −6.6 mm; and L: −2.6 mm, relative to bregma). During surgery, two small bolts were cemented in the skull to immobilize the head during the recording sessions, and a silver reference electrode was placed on the surface of the parietal cortex. The surface of the craniotomy was protected with bone wax (Ethicon, Johnson & Johnson) until recording sessions. After the surgery, mice were allowed to recover for at least 2 d. For *in vivo* recordings, the animal’s head was fixed to the recording setup, consisting of a treadmill with an infrared sensor for monitoring locomotor activity. All experiments were conducted with an amplifier (BVC-700A, Dagan Corporation) connected to a dual extracellular-intracellular headstage (8024 Dual Intracellular & Extracellular Headstage). For SEP recordings we used a micropipette with a tip diameter between 8 and 10 μm, subsequently the pipette was filled with 3 m NaCl and placed on a micromanipulator (Narishige MO-10). Whisker stimulation was performed with a pair of flexible steel electrodes (strand ø: 50.8 μm; coated ø: 228.6 μm; multistranded PFA-coated stainless-steel wire, AM Systems) inserter under the skin of the right whisker pad. The electrical stimulus consisted of a single square pulse (0.2 ms; 0.5–1 mA) delivered by an isolation unit (Cibertec ISU 210 BIP) connected to a stimulator device (CS420, Cibertec), applied every 10 ± 2 s. For single-PC activity, a micropipette with a tip diameter around 1–2 μm was filled with 3 m NaCl and placed on a micromanipulator (Narishige MO-10). The pipette was inserted in the area of interest at ∼2 μm/s, and spikes were detected based on visual (2002C and 2004C, Tektronix) and auditory cues (Audio monitor 3300, AM Systems).

Data were collected with a CED micro1401-3 data acquisition unit and sampled at 25 kHz. SEP analysis was performed with EEGLAB rev.14.1.2 toolbox using the MATLAB 2015a software package. Recorded data were segmented into 70-ms windows using the electrical stimulation as trigger and baseline was corrected by subtracting the mean voltage level in the first 20-ms interval of the window (before whisker stimulus). Data were averaged for each genotype to obtain the average SEP and temporal periods were statistically compared. For single-cell recording analysis, only well isolated neurons recorded during at least 100 s were considered. A DC remove process [time constant (s): 0.001–0.0004] was applied to reduce DC level drifts, and spikes were detected based on threshold-crossing algorithm of Spike2 software. All spikes were visually confirmed and PCs were identified by the presence of complex spikes (CSs). Subsequently, simple spikes (SSs) and CSs of each neuron were analyzed using a MATLAB custom-made script. The Predominant Firing Rate represent the mode of the firing rate, the Frequency of the firing rate that appears most often. The coefficient of variation (CV) and CV2, as a measure of firing regularity, were calculated following the formulas described previously (Holt et al., 1996) CV = $\frac{\sigma ISI}{\mu ISI}$, where ISI represents the interspike interval. CV2 = $\frac{2|ISI_{n+1}-ISI_{n}|}{\left(ISI_{n+1}+ISI_{n}\right)}$. The CSs were sorted manually offline for the duration of the recording.

### *Ex vivo* electrophysiology

Mice were anesthetized with isoflurane and decapitated. The brain was rapidly submerged in ice-cold cutting solution containing the following: 20 mm NaCl, 2.5 mm KCl, 0.5 mm CaCl_2_, 7 mm MgCl_2_, 1.25 mm NaH_2_PO_4_, 85 mm sucrose, 25 mm D-glucose, and 60 mm NaHCO_3_, saturated with 95% O_2_/5% CO_2_ (carbogen). The cerebellum was separated from the rest of the brain and the right part cut and glued to the cutting-dish. Parasagittal slices (250 μm thick) containing the Crus I area were prepared using a Campden Ci 7000smz-2 vibroslicer. Immediately on cutting, slices were submerged in an artificial CSF (aCSF) containing the following: 126 mm NaCl, 2.5 mm KCl, 1.2 mm MgCl_2_, 2.4 mm CaCl_2_, 1.2 mm NaH_2_PO_4_, 11.1 mm D-glucose, 21.4 mm NaHCO_3_, 0.1 mm ascorbic acid, and 0.4 mm kynurenic acid, bubbled with carbogen at room temperature (22–24°C), and left to recover for at least 1 h. Slices were constantly perfused with aCSF at 33°C. PCs were visualized using infrared differential interference contrast light (Olympus BX52WI). Patch pipettes pulled from thin borosilicate capillary glass (World Precision Instruments) with a Sutter P-97 horizontal puller had a resistance of 3–5 MΩ. Internal solution contained the following: 115 mm K-gluconate, 10 mm HEPES, 11 mm EGTA, 2 mm MgCl_2_, 10 mm NaCl, 2 mm MgATP, 0.25 mm Na_2_GTP, and biocytin (5 mg/ml, B4261, Sigma-Aldrich), pH 7.3, adjusted with NaOH; osmolality ± 275 mOsm.

Intrinsic properties were studied in whole-cell voltage-clamp and in current-clamp mode by injecting a hyperpolarizing bias current (<−500 pA) to hold membrane potential between −60 and −65 mV and keep the neuron silenced during the rheobase study. Membrane potentials were not corrected for the liquid junction potential between intra and external solution (−12.7 mV). Neurons in which holding current was greater than −500 pA and experiments in which access resistance was higher than 16 MΩ were discarded and not included for analysis. Signals from the patch pipette were recorded with a MultiClamp 700B amplifier, digitized at 10–20 kHz and low-pass filtered at 2–5 kHz with a Digidata 1440A analog-to-digital converter and analyzed off-line using Clampfit 10.7 software (Molecular Devices). Intrinsic excitability was determined in response to increasing depolarizing current pulses (+75 pA) of 750-ms duration injected from hyperpolarized holding currents. Rheobase was registered for each neuron as the net depolarizing current capable of inducing the first action potential (AP). PCs have been described to have different integrative properties making that neurons with similar resistances tend to respond with different firing frequencies to current pulses of similar amplitude and duration (Llinás and Sugimori, 1980). In order to facilitate the comparison between firing frequency curves, rheobase was normalized to zero and only the instantaneous frequencies between the first six APs were analyzed. AP threshold for each neuron was calculated for the first spike as the voltage where dV/dt reaches 5% of the AP maximal rise slope.

### Neuroanatomy

PCs were filled during patch-clamp recordings with biocytin (Sigma) via passive diffusion. Filled slices were fixed in 4% paraformaldehyde in PBS for 24 h at 4°C and incubated with Alexa Fluor 555/488-streptavidin (1:500) for 48 h at 4°C. Subsequently the slices were washed with PBS and mounted using Prolong Antifade Gold mounting medium (Invitrogen).

Super-resolution images were acquired using a confocal LSM880 Fast Airyscan microscope using a 25×/0.8 water (for dendrite analysis) and a 63×/0.4 oil (voxel size 49 × 49 × 211 nm, for spine analysis) Plan-Apochromat objectives. Sholl analysis and spine quantification were performed using Fiji ImageJ software. For Sholl, the number of intersections of the dendritic arbour with concentric circles drawn at 5-μm intervals from the soma was counted. Spines were manually counted along a 10-μm distal dendrite.

### Statistical analysis

Data are displayed through the graphs as mean ± SEM. Statistical analyses were performed using MATLAB (The MathWorks, Inc.) and Prism (GraphPad). For comparisons between groups a Student’s *t* test or two-way ANOVA with repeated measures, when appropriate, were used, as indicated in each figure legend. The significance threshold was set at *p *=* *0.05 (ns = not significant, *p <* *0.05, **p <* *0.01, ***p <* *0.001). Effect sizes as denoted by mean differences (MDs) are displayed on the right of the graphs, when appropriate. Estimation statistics based on 95% confidence interval (CI) of the MD is shown. Plots were performed with https://www.estimationstats.com. Artistic figures were created with Biorender.

## Results

### Spontaneous *in vivo* PC activity is altered in *Cntnap2*−/− mice

**Figure 1.**
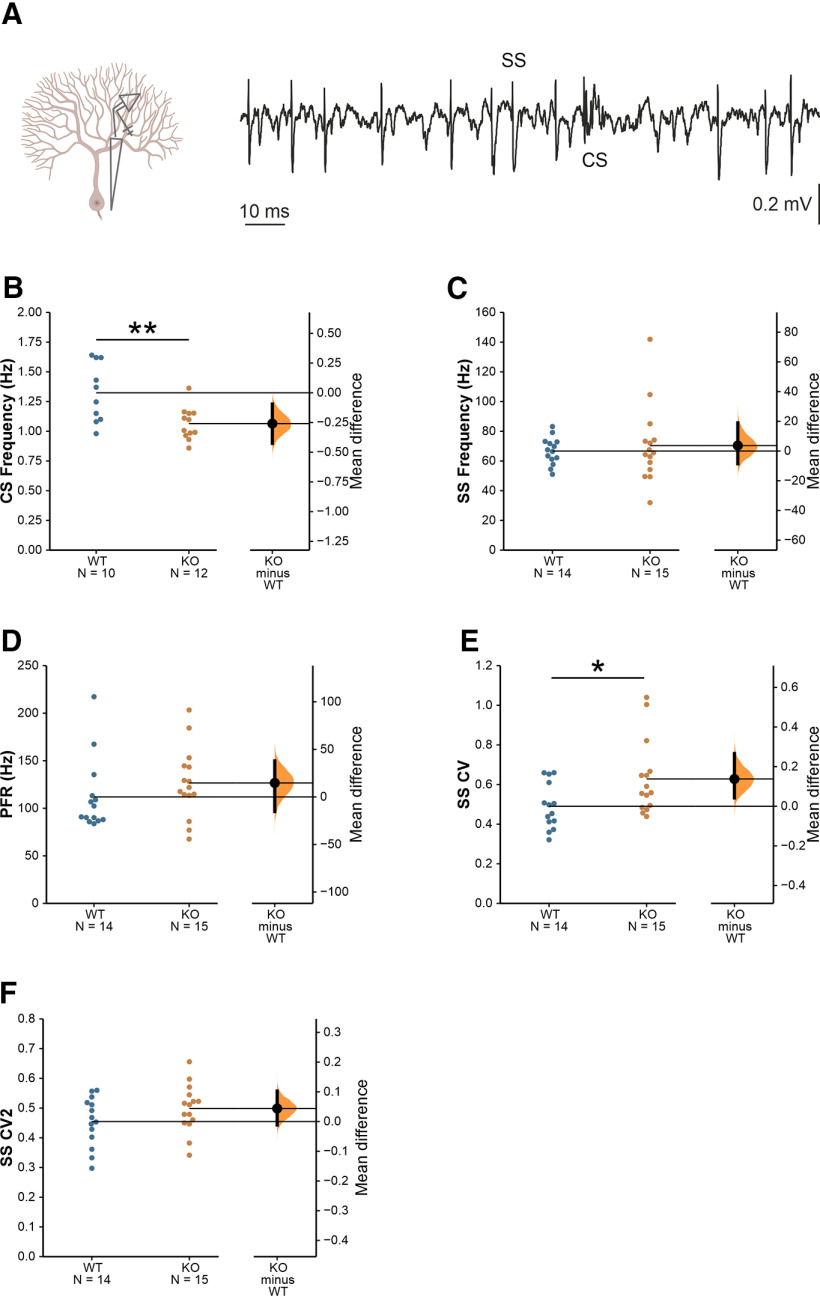
*Cntnap2* mice show altered *in vivo* spontaneous activity of PCs. ***A***, Schematic illustration of an extracellular PC recording and a representative trace displaying SSs and CSs. ***B***, CS firing rate, MD −0.259 [95.0% CI −0.426, −0.0951], Student’s *t* test *p *=* *0.0054. ***C***, SS firing rate, MD 3.68 [95% CI −8.55, 19.0], Student’s *t* test *p *=* *0.656. ***D***, SS predominant firing rate, MD 14.7 [95.0% CI −15.4, 37.9], Student’s *t* test *p *=* *0.31. ***E***, CV of the interspike intervals for SS, MD 0.138 [95.0% CI 0.0422, 0.266], Student’s *t* test *p *=* *0.0242. ***F***, CV for adjacent interspike intervals (CV2), MD 0.0439 [95.0% CI −0.0117, 0.103], Student’s *t* test *p *=* *0.153. Data are presented as mean ± SEM. *N* = 14 WT, 15 KO neurons. MD = mean difference (KO-WT); CI, confidence interval; *p <* *0.05, **p <* *0.01.

We first performed extracellular recordings of spontaneous PC activity in the Crus I/II area of awake animals (Fig. 1*A*). Crus I/II was selected because of its functional relationship with sensory whisker inputs (Gao et al., 1996) and its association with autism (Skefos et al., 2014; D’Mello et al., 2016; Wang et al., 2020). PCs present two types of firing patterns: SSs and CSs, generated by distinct inputs (parallel fibers and climbing fibers, respectively) and distinguished by their particular waveforms. Despite their different origin interactions between the two types of spikes have been described, such that CS firing is thought to modulate SS activity (Tang et al., 2017). We found a lower CS firing frequency in *Cntnap2* KO mice compared with WT controls (Fig. 1*B*). The firing frequency of SSs (Fig. 1*C*), as well as the predominant, or preferred, SS firing frequency (Fig. 1*D*) were not different between genotypes. However, *Cntnap2* KO mice show a higher irregularity in the temporal firing pattern of SSs (Fig. 1*E*), as measured by the CV of the interspike intervals. Such difference is not observed when adjacent interspike intervals of presumably burst firing are considered, denoted by CV2 (Fig. 1*F*).

### PC responses to sensory-evoked stimuli are altered in *Cntnap2*−/− mice

**Figure 2.**
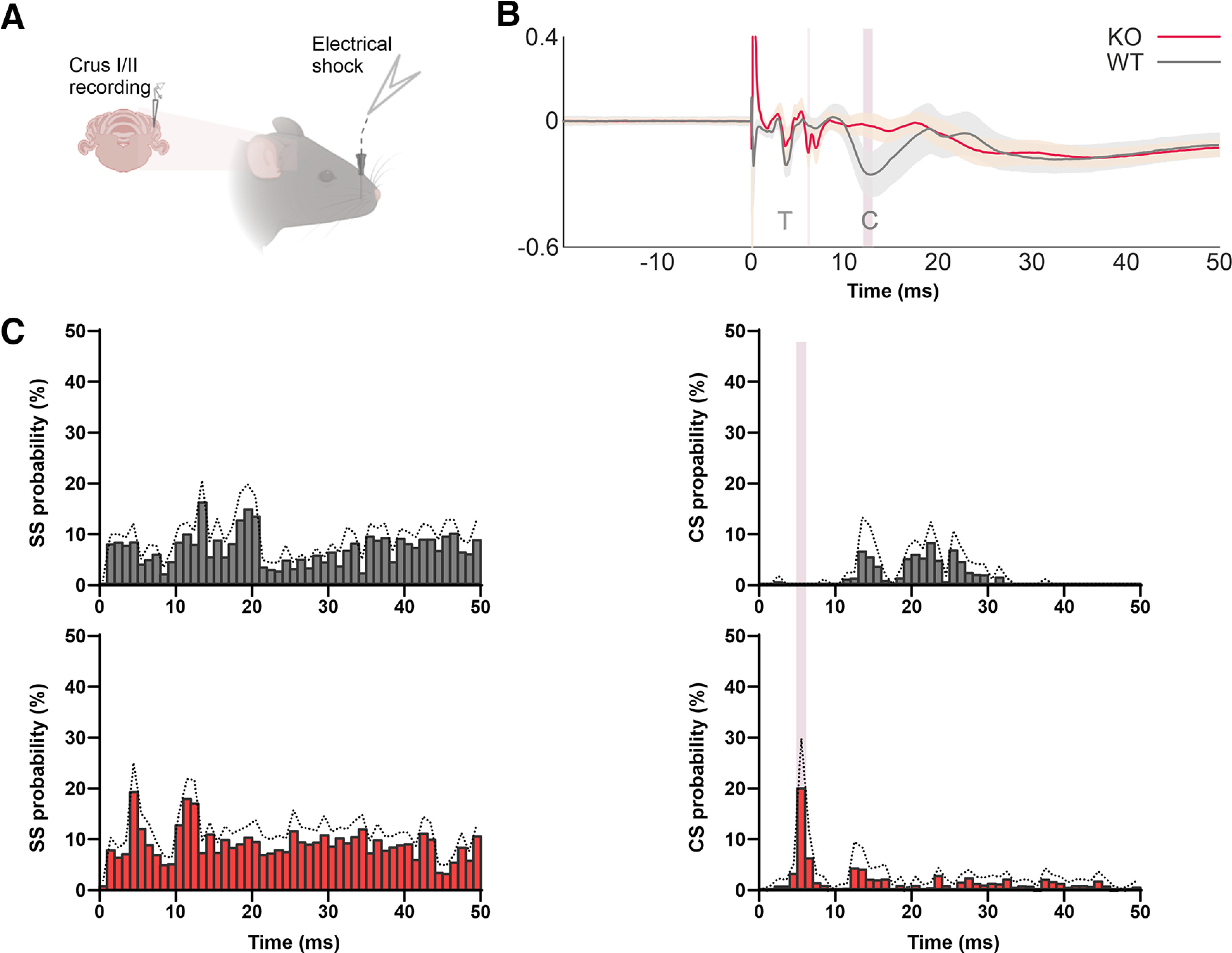
Altered cerebellar LFP after electrical stimulation of the whisker pad in *Cntnap2* KOs. ***A***, Schematic illustration representing the LFP recording. ***B***, Event-related potential analysis comparing the average SEP traces for nine KO (red trace) and six WT (black trace) mice. Vertical pink bars indicate statistically different latencies, corresponding to the cortical peak (C) in WT at 12.87 ± 0.40 ms, which is absent in KOs, who show a statistically significant novel negative peak, presumably an anticipated cortical response, at 6.46 ± 0.14 ms. Student’s *t* test (*p *<* *0.05). ***C***, Temporal firing pattern for SS (left) and CS (right) in WT (*N* = 6 neurons, black) and KO (*N* = 10 neurons, red) mice after electrical stimulation. Note that CS appear at poststimulation latencies concordant with the C component in WT, while they appear earlier in KO mice, likely indicating an anticipated C response at 6 ms. Two-way ANOVA, Holm–Sidak test for multiple comparisons (*p *<* *0.0001). The graphs represent the mean ± SEM (discontinuous or shaded lines).

To characterize PC activity in response to somatosensory stimuli, we recorded the local field potential (LFP) near the PC layer from the cerebellar Crus I/II area after subcutaneous electrical stimulation of the ipsilateral whisker pad in alert mice (Fig. 2*A*).This protocol induces a similar but more reproducible response than tactile whisker stimulation (Márquez-Ruiz and Cheron, 2012).

Concordant with what was previously described (Márquez-Ruiz and Cheron, 2012), in WT mice this electrical stimulation evoked a highly reproducible sensory evoked potential (SEP) with two main negative components appearing at around 4 and 12 ms (3.94 ± 0.13 ms and 12.87 ± 0.40 ms), corresponding to trigeminal (T) and cortical (C) pathways, respectively. Strikingly, the waveform of SEP in *Cntnap2* KO mice was notably different, showing the expected T wave (4.10 ± 0.15 ms) and a novel negative peak at around 6 ms (6.46 ± 0.14 ms) in the absence of the expected C wave at 12 ms (Fig. 2*B*). To determine the basis for the altered SEP response observed in *Cntnap2*−/− mice, we then recorded unitary PC upon whisker stimulation. Previous studies have correlated the appearance of a SS burst with the above-mentioned T component, whereas CS occurred at poststimulation latencies concordant with the C component (Mostofi et al., 2010; Márquez-Ruiz and Cheron, 2012). In agreement with this, we found that the probability for a CS to fire around 13 ms (12.87 ms) after the stimulus onset in WT mice is coincident with the C wave observed in the SEP. In *Cntnap2*−/− mice, however, the bigger probability for a CS to appear takes place at 6.46 ms, matching the latency of the novel peak observed in the corresponding SEP (Fig. 2*C*). As for SS firing, in both genotypes we observed a slight decrease in SS firing probability just after the CS occurrence, between 15–18 ms for WT and 6–10 ms for KO mice, but these differences were not statistically significant. Although the precise origin of this novel peak needs to be elucidated, the data suggest that the cerebellar representation of cortical input is anticipated in *Cntnap2*−/− mice.

### Increased PC intrinsic excitability in *Cntnap2* KO mice

**Figure 3.**
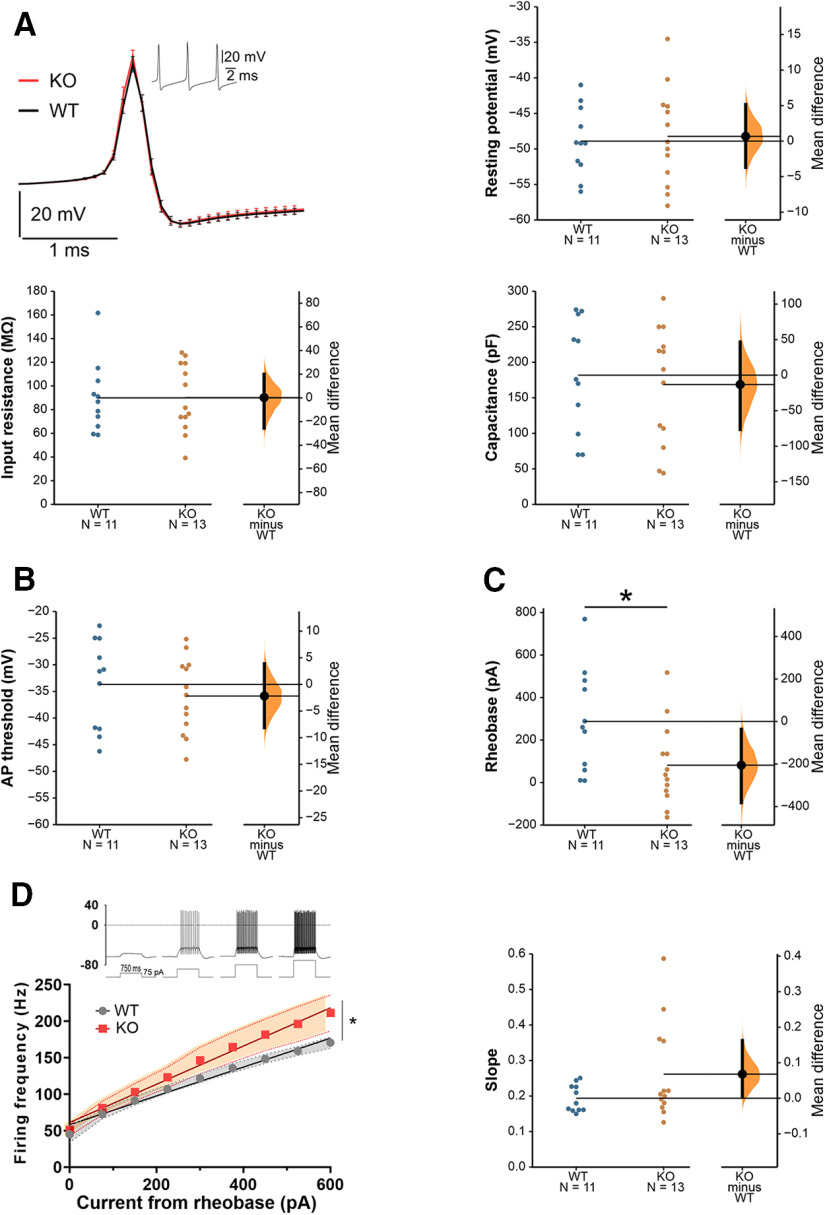
Increased intrinsic excitability of PCs in *Cntnap2* KOs. ***A***, Representative AP from WT and KO mice. No significant differences were found in passive membrane properties between WT and KO PC. Resting potential MD 0.6720 [95.0% CI −3.7, 5.15], Student’s *t* test *p *=* *0.778. Input resistance MD 0.263 [95.0% CI −25.5, 20.1], Student’s *t* test *p *=* *0.986. Capacitance MD −13.22 [95.0% CI −76.5, 46.7], Student’s *t* test *p *=* *0.691. ***B***, AP threshold MD −2.180 [95.0% CI −8.12, 3.88], Student’s *t* test *p *=* *0.499. ***C***, Rheobase MD −205 [95.0% CI −382, −36.4], Student’s *t* test *p *=* *0.034. ***D***, Intrinsic excitability as a measurement of firing frequency on current step increases (left) and individual slopes of the frequency/intensity lines (right). Two-way ANOVA mixed effect (interaction genotype × current *p *=* *0.03), slopes MD 0.0679 [95.0% CI 0.00631, 0.163], Student’s *t* test *p *=* *0.12. All data are presented as mean ± SEM. *N* = 11 WT, 13 KO neurons. MD = mean difference (KO-WT); CI, confidence interval; *p <* *0.05. Membrane potentials are not corrected for the liquid junction potential between intra and external solution (−12.7 mV).

To assess whether alterations in the intrinsic properties of PCs could be responsible for the aberrant firing found during spontaneous or evoked activity, we performed whole cell patch-clamp recordings of PCs from the Crus I/II area in sagittal cerebellar slices. No differences were found in passive membrane properties (resting potential, input resistance, membrane capacitance) between WT and *Cntnap2* KO mice (Fig. 3*A*). Similarly, no differences in AP threshold were found between genotypes (Fig. 3*B*). The injected current needed to evoke the first AP (rheobase) was smaller in *Cntnap2* KOs (Fig. 3*C*), suggesting increased excitability. To specifically study PC intrinsic excitability, we measured the neuronal firing frequency elicited by depolarizing current steps of increasing amplitude. Both genotypes showed a linear current/frequency increase, as previously described for PCs (Llinás and Sugimori, 1980; Fernandez et al., 2007) but, as expected based on their smaller rheobase, PCs in *Cntnap2* KO mice fired at a higher frequency for the same injected current, indicating higher excitability (Fig. 3*D*). The slopes of the individual current/frequency lines of the neurons for each genotype were also analyzed showing that a subgroup of neurons seem to be driving the observed excitability effects, supporting the increased data variability generally observed in Cntnap2 KOs.

### Reduced dendritic complexity of PCs in *Cntnap2* mice

**Figure 4.**
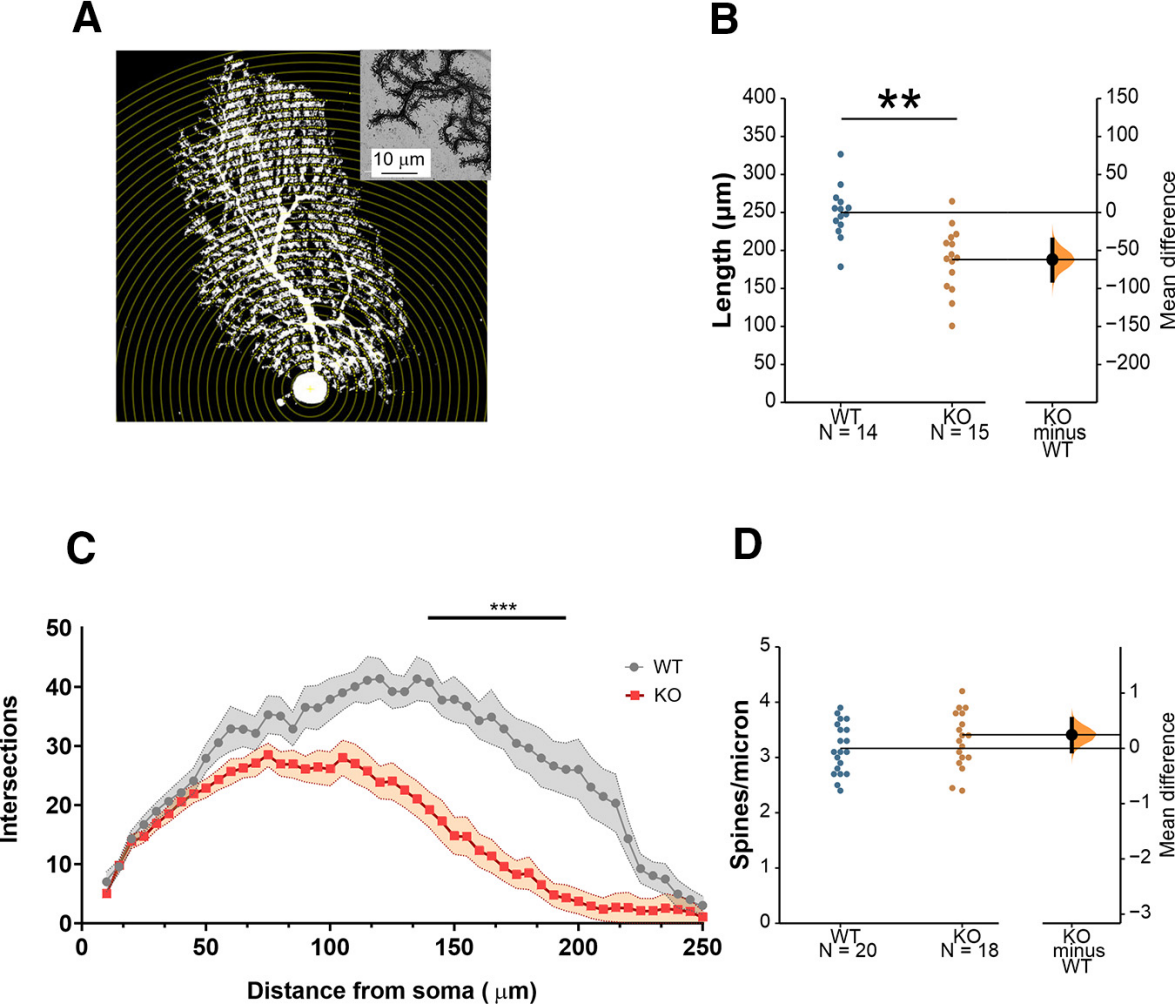
Reduced arborization of PCs in *Cntnap2* KOs. ***A***, Representation of Sholl analysis applied to a PC. ***B***, PC length from soma to the most apical point, MD −61.89 [95.0% CI −89.6, −35.7], Student’s *t* test *p *=* *0.0002. *N* = 14 WT, 15 KO. ***C***, Number of intersections as a function of their distance from the soma. Two-way ANOVA with repeated measures. *N* = 11 WT, 16 KO. ***D***, Spine density MD 0.246 [95.0% CI −0.0533, 0.532], Student’s *t* test *p *=* *0.117. *N* = 20 WT, 18 KO. Data are presented as mean ± SEM. MD = mean difference (KO-WT); CI, confidence interval; **p <* *0.01, ***p <* *0.001.

Recently, *Cntnap2* has been reported to modulate the development of PC (Argent et al., 2020). Further, we previously demonstrated that *Cntnap2* KO mice show defects in spine stabilization in the cerebral cortex (Gdalyahu et al., 2015). To determine whether the electrophysiological alterations observed in PCs in *Cntnap2* KOs are associated with morphologic changes, we characterized PC morphology in the patched neurons by biocytin staining (Fig. 4*A*). We found that the overall length of the cells is significantly smaller in *Cntnap2* mice (Fig. 4*B*). Sholl analysis of dendritic complexity demonstrated that PCs of KO mice not only are smaller but also have a less complex dendritic arbor (Fig. 4*C*). Last, no statistically significant differences in spine density were found between genotypes (Fig. 4*D*).

## Discussion

There is growing evidence for the involvement of the cerebellum in ASD through its role in integrating sensory neural signals. In this work, we investigated sensory processing in the cerebellum *in vivo* in the *Cntnap2* mouse model of autism, an autism-linked gene involved in cerebellar development and function. We used a well-characterized paradigm to study cerebellar processing of sensory function, where the evoked activity of PCs, the sole output of the cerebellum, is analyzed upon whisker stimulation (Cheron et al., 2013). Whisker-related sensory information is conveyed to the cerebellar area Crus I/II, an area widely associated with ASD (Fernandez et al., 2019). We found that the evoked response of Crus I/II PCs to sensory stimuli (electrical stimulation of the whisker pad) was strikingly different between WT and *Cntnap2* KO mice, as denoted by the generated SEP. Whisker information reaches the cerebellum via two routes, the pontine nuclei-parallel fiber pathway and the inferior olive-climbing fiber pathway (Kleinfeld et al., 1999), generating the two distinct types of firing that characterize PCs: SSs and CSs, respectively. Individual analysis of each type of spike upon stimulation revealed that the timing of appearance of the stimulus-evoked CS, anticipated in *Cntnap2* KOs, was driving the observed differences in SEP, indicating an altered processing of climbing fiber inputs. In agreement with this, dysfunction of the olivocerebellar circuit in *Cntnap2* mice was proposed as responsible for the decreased response probability shown by this model in the eye-blink conditioning test, another paradigm widely used to study cerebellar function (Kloth et al., 2015). The appearance of the stimulus-evoked CS has been shown to be modulated by activity from the somatosensory cortex (S1), since the suppression of S1 activity abolishes CS appearance upon tactile stimulation (Shimuta et al., 2020). Further, diminishing S1 activity lengthens and enhancing it shortens CS appearance latency (Brown and Bower, 2002). These data would suggest that an increased activity of S1 could be responsible for the observed CS anticipation in the *Cntnap2* model. In fact, decreased inhibitory markers and increased excitation/inhibition ratio leading to higher cortical sensory gain have been described in *Cntnap2* mice (Peñagarikano et al., 2011; Antoine et al., 2019), which could account for the observed deficits.

CSs have been recently shown to control the information encoded by SS activity (Streng et al., 2017) in a way that a dynamic relationship between CSs and SSs is necessary for the initiation of sensory-driven behavior (Tsutsumi et al., 2020). We observed reduced spontaneous CS firing frequency as well as increased irregularity in the SS timing, despite normal SS firing frequency. The rhythmicity of PC interspike intervals is thought to affect the transmission of information to downstream neurons. It is worth noting that increased CV has been reported in other animal models of autism with cerebellar dysfunction including *CACNA1A* (Damaj et al., 2015; Jayabal et al., 2016), *CAMK2B* (van Woerden et al., 2009; Küry et al., 2017), and *KCNMA1* (Laumonnier et al., 2006; Chen et al., 2010), the last two being also associated with a reduced CS firing frequency. In the adult stage, each PC receives strong excitatory inputs through a single climbing fiber from the inferior olive, which innervates several PCs, generating CS activity. As *CNTNAP2* has been shown to be expressed not only in PCs, but also in inferior olivary neurons (Kloth et al., 2015), alterations in these neurons could likely change the baseline firing rate of CSs and could in turn affect the regularity of SSs.

Given the observed spontaneous PC firing in vivo, the observation of increased PC excitability and reduced arborization in *Cntnap2* KOs was unexpected. It must be noted that normal PC arborization was described for this same model (Kloth et al., 2015). One possible explanation for this discrepancy is methodological differences (Golgi stain vs neuronal filling) or more likely a different cerebellar area was analyzed. In fact, it has been recently shown that the cerebellar cortex is non-uniform and that several features of PCs, including activity levels and dendritic arborization develop differentially based on the specific cerebellar area where they are located (Beekhof et al., 2021). On the other hand, voltage-gated potassium (Kv) channels, such as Kv3.3, have been involved in regulating CSs. CNTNAP2 is known to cluster Kv channels, including KCNA1 (Kv1.1) and KCNA2 (Kv1.2; Poliak et al., 1999). Reduced expression of such channels was observed in hippocampal tissue resected from epileptic patients harboring *CNTNAP2* mutations (Strauss et al., 2006). Whether the observed alterations in PC excitability are due to reduced expression or mislocalization of Kv channels and its contribution to circuit dysfunction remains to be investigated.

In summary, we report an altered cerebellar response to evoked sensory stimuli in alert *Cntnap2* mice. This alteration is found in conjunction with neuroanatomical and electrophysiological dysfunction of PCs. This mouse model, therefore, provides a valuable tool to study the basis for the altered processing of sensory information observed in ASD.

## Synthesis

Reviewing Editor: David Schoppik, New York University - Langone Medical Center Decisions are customarily a result of the Reviewing Editor and the peer reviewers coming together and discussing their recommendations until a consensus is reached. When revisions are invited, a fact-based synthesis statement explaining their decision and outlining what is needed to prepare a revision will be listed below.

Traditionally eNeuro decision letters include a full summary of reviews and selected comments. I’m not including one here because the revised manuscript and rebuttal letter adequately treats the original reviewers’ suggestions and comments. Please change the following three things:

1) The versions of Figure 2 differ between the “Merged File” and “Clean Copy;” please keep the one from the “Merged File” unless there’s some reason not to?

2) Please remove the bars from Figures 1B-1F and Figure 4 to be clearer and consistent with Figure 3.

3) Please add the text about the ∼12mV liquid junction potential to the legend of Figure 3 to avoid future confusion.
